# Antibody Response to Symptomatic Infection With SARS‐CoV‐2 Omicron Variant Viruses, December 2021–June 2022

**DOI:** 10.1111/irv.13339

**Published:** 2024-07-16

**Authors:** Ryan Sandford, Ruchi Yadav, Emma K. Noble, Kelsey Sumner, Devyani Joshi, Sara Y. Tartof, Karen J. Wernli, Emily T. Martin, Manjusha Gaglani, Richard K. Zimmerman, H. Keipp Talbot, Carlos G. Grijalva, Edward A. Belongia, Christina Carlson, Melissa Coughlin, Brendan Flannery, Brad Pearce, Eric Rogier

**Affiliations:** ^1^ Centers for Disease Control and Prevention Atlanta Georgia USA; ^2^ Oak Ridge Institute for Science and Education Oak Ridge Tennessee USA; ^3^ Rollins School of Public Health Atlanta Georgia USA; ^4^ Department of Research & Evaluation Kaiser Permanente Southern California Yorba Linda California USA; ^5^ Department of Health Systems Science Kaiser Permanente Bernard J. Tyson School of Medicine Pasadena California USA; ^6^ Kaiser Permanente Washington Health Research Institute Seattle Washington USA; ^7^ University of Michigan School of Public Health Ann Arbor Michigan USA; ^8^ Baylor Scott & White Health Temple Texas USA; ^9^ Texas A&M University College of Medicine Temple Texas USA; ^10^ University of Pittsburgh Pittsburgh Pennsylvania USA; ^11^ Vanderbilt University Medical Center Nashville Tennessee USA; ^12^ Marshfield Clinic Research Institute Marshfield Wisconsin USA

**Keywords:** COVID‐19, immune response, Omicron subvariants, SARS‐CoV‐2 infection

## Abstract

We describe humoral immune responses in 105 ambulatory patients with laboratory‐confirmed SARS‐CoV‐2 Omicron variant infection. In dried blood spot (DBS) collected within 5 days of illness onset and during convalescence, we measured binding antibody (bAb) against ancestral spike protein receptor binding domain (RBD) and nucleocapsid (N) protein using a commercial multiplex bead assay. Geometric mean bAb concentrations against RBD increased by a factor of 2.5 from 1258 to 3189 units/mL and by a factor of 47 against N protein from 5.5 to 259 units/mL between acute illness and convalescence; lower concentrations were associated with greater geometric mean ratios. Paired DBS specimens may be used to evaluate humoral response to SARS‐CoV‐2 infection.

## 1 Introduction

Humoral immune responses to infection with SARS‐CoV‐2 include production of immunoglobulin G (IgG) antibodies that bind to spike (S) glycoprotein, including the receptor binding domain (RBD) within S, as well as the nucleocapsid (N) protein. Virus neutralization titers and S protein binding antibodies have been associated with protection against symptomatic infection with ancestral and pre‐Omicron SARS‐CoV‐2 variants [1, 2, 3, 4]. Because antibodies to N protein are not elicited by US‐licensed COVID‐19 vaccines, the presence of anti‐N antibodies can be an indicator of past SARS‐CoV‐2 infection among vaccinated and unvaccinated individuals [5, 6]. Elevated levels of anti‐N antibody may indicate more recent SARS‐CoV‐2 infection [7]. As new SARS‐CoV‐2 variants have emerged, serologic assays that quantify IgG antibody binding to S and N protein antigens have been used to evaluate humoral response to SARS‐CoV‐2 infection [8]. Postinfection antibodies may reduce risk of reinfection with new SARS‐CoV‐2 variants, suppress viral replication, and reduce COVID‐19 disease severity following reinfection [2, 8].

DBS specimens have been validated as alternatives to venous blood for use with multiple SARS‐CoV‐2 binding antibody assays [9, 10, 11]. Using DBS specimens collected during acute illness from patients with laboratory‐confirmed COVID‐19 and non‐COVID‐19 illnesses, we previously showed that anti‐N antibody seropositivity modified COVID‐19 mRNA vaccine effectiveness against symptomatic SARS‐CoV‐2 infection with SARS‐CoV‐2 Delta and Omicron variants [12]. Among acutely ill patients, higher levels of binding antibodies against ancestral S protein reduced the odds of testing positive for SARS‐CoV‐2 Delta and Omicron variants [13]. Here, we assessed humoral immune response to SARS‐CoV‐2 infection comparing acute‐ and convalescent‐phase IgG antibody levels against N and ancestral S RBD antigens among case patients infected during Omicron variant–predominant periods from December 2021 through June 2022.

Respiratory swabs and acute‐phase DBS were obtained < 5 days after symptom onset from ambulatory patients with respiratory illness enrolled in the US Influenza Vaccine Effectiveness Network [12, 13]. Previous analysis demonstrated no increase in mean anti‐N antibody concentration from DBS collected < 5 days after symptom onset among SARS‐CoV‐2‐infected patients [13]. Patients who tested positive for SARS‐CoV‐2 by nucleic acid amplification in respiratory specimens were scheduled for convalescent‐phase blood sample collection at 21–56 days after enrollment. Data collected from enrolled participants included patient age, date of illness onset, symptoms associated with COVID‐19‐like illness, self‐reported presence of specified underlying medical conditions, documented COVID‐19 vaccination dates, self‐reported laboratory‐confirmed COVID‐19 < 90 or ≥ 90 days before current illness and electronic medical record of positive COVID‐19 tests.

Methods for estimating SARS‐CoV‐2 bAb concentration from DBS have been previously published [11]. DBS specimens were tested for IgG antibodies against SARS‐CoV‐2 recombinant antigens representing ancestral S protein RBD and N protein using a validated multiplex bead assay (FlexImmArray™ SARS‐CoV‐2 Human IgG Antibody Test, Tetracore, Rockville, MD) on a Luminex MAGPIX instrument with LX200 flow analyzer (Luminex Corporation, Austin, TX) [14]. Eluted specimens were diluted 1:300, and individual specimen median fluorescence intensity (MFI) ratios were calculated compared to antigen‐specific human IgG calibrator serum. MFI units were standardized to binding antibody unit per milliliter (BAU/mL) against World Health Organization (WHO) international standards [9, 13].

Geometric mean bAb concentration (GMC), geometric mean ratios of bAb concentrations at follow‐up versus enrollment, and 95% confidence interval (CI) were calculated on the log_10_ scale and back‐transformed to original units. We interpret geometric mean ratios as the magnitude of an immune response to ancestral S RBD and N protein following natural infection. Associations between mean bAb ratios and patient characteristics, vaccination status, baseline N antibody serostatus, and prior positive COVID‐19 test were assessed by *t*‐test or ANOVA. Statistical analysis was performed using R version 4.0.3 (R Foundation for Statistical Computing, Vienna, Austria).

A total of 105 SARS‐CoV‐2‐positive participants had acute‐phase blood specimens collected a median of 2 days (range: 0–5) and convalescent‐phase specimens collected a median of 36 days (range: 23–61) after symptom onset. Of 48 SARS‐CoV‐2‐positive specimens with genomic sequencing, 43 (90%) belonged to Omicron BA.1 (*n* = 5) or BA.2 (*n* = 38) lineages, with five sequences belonging to BA.2.12.1 (*n* = 3), BA.4 (*n* = 1), and BA.5 (*n* = 1) lineages. Of 105 case patients, 36% were aged < 40 years, 17% were > 65 years, 57% were female, and 23% had an underlying health condition (Table 1). Among 7 (7%) patients with ≥ 1 electronic health record documented prior COVID‐19 positive laboratory PCR test, median time since most recent positive COVID‐19 test was 351 days (range: 64–758). Among 101 patients who had received at least two doses of COVID‐19 mRNA vaccine, median time since receipt of most recent mRNA vaccine dose was 175 days (range: 33–382); 11 had received a booster dose < 90 days prior to their enrollment in the study (measured between the date of booster and date of illness onset).

**TABLE 1:** Concentrations and mean increase in binding antibodies against SARS‐CoV‐2 nucleocapsid and ancestral spike receptor binding domain antigens during acute‐ and convalescent‐phase of symptomatic infection during Omicron‐predominant variant period.

	Total *N* = 105 (%)	α‐SARS‐CoV‐2 spike receptor binding domain antibody				α‐SARS‐CoV‐2 nucleocapsid antibody			
		Geometric mean concentration, BAU/mL (95% CI)		Geometric mean ratio (95% CI)	*p* value^a^	Geometric mean concentration, BAU/mL (95% CI)		Geometric mean ratio (95% CI)	*p* value^a^
		Acute	Convalescent			Acute	Convalescent		
**Overall**	105	1257.8 (923.9, 1712.3)	3188.5 (2638.7, 3853.0)	2.5 (1.9, 3.3)		5.5 (4.3, 7.1)	259.4 (200.6, 335.4)	47.0 (33.5, 65.9)	
**Age group**					0.867				0.815
0–39 years	38 (36)	1131.0 (618.4, 2068.7)	2837.9 (2091.0, 3851.5)	2.5 (1.4, 4.6)		5.0 (3.4, 7.4)	256.9 (160.2, 412.0)	51.0 (28.0, 93.0)	
40–65 years	49 (47)	1374.4 (965.9, 1955.6)	3706.1 (3147.8, 4363.4)	2.7 (1.9, 3.8)		6.7 (4.3, 10.5)	279.7 (193.9, 403.3)	41.8 (24.7, 70.9)	
> 65 years	18 (17)	1236.4 (463.3, 3299.5)	2707.5 (1154.6, 6349.4)	2.2 (1.3, 3.6)		4.0 (2.9, 5.6)	215.7 (113.7, 409.3)	54.1 (27.1, 107.8)	
**Sex**					0.350				0.580
Female	60 (57)	1080.7 (661.6, 1765.2)	3058.5 (2304.3, 4059.4)	2.8 (1.9, 4.1)		4.8 (3.0, 7.7)	222.7 (130.2, 380.8)	46.6 (24.6, 88.0)	
Male	45 (43)	1539.8 (1124.3, 2108.8)	3370.6 (2648.0, 4290.3)	2.2 (1.5, 3.3)		5.6 (4.1, 7.8)	273.7 (199.5, 375.6)	48.5 (31.6, 74.4)	
**Underlying medical condition** ^b^					0.770				0.914
Yes	24 (23)	1505.0 (995.7, 2274.6)	3705.4 (3042.7, 4512.3)	2.5 (1.6, 3.8)		4.8 (3.0, 7.7)	222.7 (130.2, 380.8)	46.6 (24.6, 88.0)	
No	76 (72)	1112.0 (742.0, 1666.3)	2970.4 (2303.4, 3830.4)	2.7 (1.9, 3.8)		5.6 (4.1, 7.8)	273.7 (199.5, 375.6)	48.5 (31.6, 74.4)	
**COVID‐19 vaccination status**					**< 0.001**				0.525
Unvaccinated	4 (4)	16.0 (0.2, 1556.6)	149.0 (2.4, 9353.8)	9.3 (0.3, 273.1)		9.8 (0.8, 125.0)	1133.6 (631.7, 2034.2)	115.5 (6.7, 1998.0)	
2 mRNA doses	14 (13)	414.6 (148.1, 1160.5)	3268.3 (2293.0, 4658.4)	7.9 (2.6, 24.3)		3.9 (1.3, 11.9)	214.3 (120.5, 380.9)	55.2 (17.6, 173.2)	
≥ 3 mRNA doses	87 (83)	1837.7 (1473.7, 2291.6)	3656.2 (3190.6, 4189.7)	2.0 (1.6, 2.5)		5.7 (4.4, 7.3)	250.0 (186.8, 334.5)	43.9 (30.4, 63.4)	
**Baseline α‐SARS‐CoV‐2 nucleocapsid antibody serostatus** ^c^					**0.001**				**< 0.001**
Seropositive^e^	33 (31)	2065.3 (1211.5, 3520.9)	2685.7 (1881.4, 3834.1)	1.3 (0.8, 2.1)		24.0 (15.4, 37.4)	350.5 (198.2, 620.0)	14.6 (7.0, 30.3)	
Seronegative	72 (69)	1002.0 (689.3, 1456.8)	3449.4 (2750.9, 4325.3)	3.4 (2.5, 4.7)		2.8 (2.4, 3.2)	226.0 (171.8, 297.1)	80.3 (59.4, 108.5)	
**Prior COVID‐19 positive test** ^d^					0.7100				0.754
Yes^e^	7 (7)	1476.3 (326.9, 6665.9)	2928.8 (1414.9, 6062.7)	2.0 (0.8, 4.9)		33.1 (7.1, 153.8)	983.4 (545.9, 1771.2)	29.7 (4.5, 196.5)	
No	98 (93)	1243.5 (902.3, 1713.5)	3207.9 (2628.6, 3914.9)	2.6 (1.9, 3.4)		4.9 (3.8, 6.2)	235.8 (181.2, 307.0)	48.5 (34.3, 68.7)	

Abbreviation: BAU, binding antibody unit.

^a^ Student *t*‐test or ANOVA test of difference between log transformed geometric mean ratio; *p* < 0.05 defined as statistically significant difference between groups.

^b^ Self‐reported presence of a serious chronic medical condition such as heart disease, lung disease, diabetes, cancer, liver or kidney disease, immune suppression, or high blood pressure. Missing *n* = 5.

^c^ Seropositivity (nucleocapsid BAU/mL ≥ 6.9) based on conversion from mean fluorescence intensity ratio ≥ 1.2 of sample to calibrator serum in multibead assay to BAU/mL using WHO standard serum panel.

^d^ Prior COVID‐19 positive test indicates electronic medical record documentation of prior positive RT‐PCR SARS‐CoV‐2 test results; no prior test indicates that no positive RT‐PCR SARS‐CoV‐2 test was documented in electronic health records.

^e^ Nucleocapsid seropositivity and/or prior COVID‐19 positive test considered evidence of prior SARS‐CoV‐2 infection (34 patients; 32%).

Anti‐N and anti‐RBD geometric mean ratio increases were not associated with days from symptom onset to specimen collection. For both antigens, geometric mean ratios from acute‐ to convalescent‐phase bAb concentrations were similar by patient age, sex, or presence of underlying medical conditions.

**FIGURE 1:**
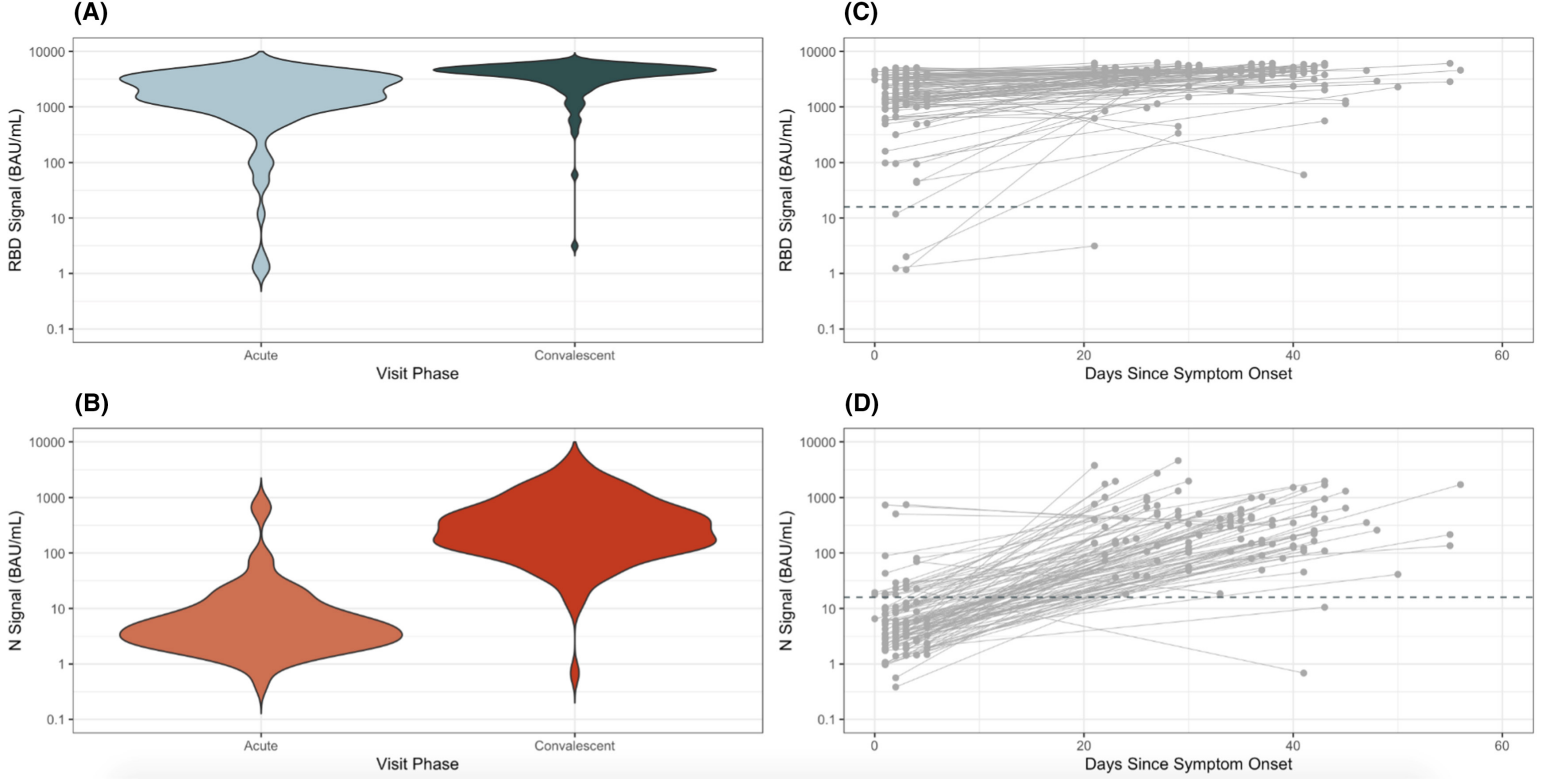
Concentrations of antibodies against SARS‐CoV‐2 nucleocapsid (N) and ancestral spike protein receptor binding domain (RBD) antigens during acute and convalescent phases of symptomatic COVID‐19 associated with Omicron variant virus infection, December 2021–June 2022. (A) Ancestral spike protein receptor binding domain (RBD) observed in acute‐ and convalescent‐phase dried blood spot specimens collected from individuals with symptomatic COVID‐19 and (B) antibody concentrations (in binding antibody unit [BAU]/mL) against SARS‐CoV‐2 nucleocapsid protein (N). (C) RBD concentration in days since symptom onset between acute and convalescent specimens from individuals with symptomatic COVID‐19; acute‐ and convalescent‐phase specimens from the same individual (*N* = 105) are connected by solid gray line and (D) changes in N concentration.

Geometric mean anti‐S RBD bAb concentrations were 1258 (CI: 924–1712) and 3189 (CI: 2639–3853) in acute‐ and convalescent‐phase specimens, respectively; mean RBD bAb ratio was 2.5 (CI: 1.9–3.3; Table 1 and Figure 1). Geometric mean ratio in RBD bAb was higher among patients who had received two mRNA vaccine doses (7.9 [CI: 2.6—24.3]) versus ≥ 3 doses (2.0 [CI: 1.6–2.5]). Acute‐ and convalescent‐phase anti‐RBD bAb levels were low among four unvaccinated case patients (GMC: 16.0 [CI: 0.2–1557] acute and 149.0 [CI: 0.3–273.1] convalescent).

In contrast, anti‐N bAb concentrations in acute‐phase specimens were low (GMC: 5.5 BAU/mL, 95% CI: 4.3–7.1; Figure 1); 72 (69%) participants had anti‐N bAb concentrations below the seropositivity threshold of 6.9 BAU/mL. From enrollment to follow‐up, anti‐N bAb concentration increased by a factor of 47 (CI: 34–66), with convalescent‐phase GMC of 259 BAU/mL (CI: 201–335). Among participants with anti‐N bAb levels below 6.9 BAU/mL at enrollment, concentrations increased by a factor of 80 (CI: 59–109), versus a factor of 15 (CI: 7–30) among participants with acute‐phase concentrations ≥ 6.9 BAU/mL (*p* value = 0.007). Among four unvaccinated case patients, anti‐N bAb concentrations increased by a factor of 116 (95% CI: 7–1998). Patients with prior documented positive COVID‐19 tests trended towards having higher convalescent‐phase anti‐N bAb concentrations than patients without prior positive COVID‐19 tests (GMC: 983 [95% CI: 546–1771] vs. 236 [95% CI: 181–307]).

Increased convalescent anti‐N bAb levels among baseline seropositive patients suggested boosting of immune responses acquired from past infection; this boosting was also observed among patients with ≥ 1 documented prior COVID‐19 positive test. In contrast, lower baseline anti‐RBD antibody concentrations among patients who had received 2 versus ≥ 3 mRNA vaccine doses were associated with greater increase in antibody response but similar convalescent antibody concentrations. While anti‐RBD bAb levels correlate with protection from infection with pre‐Omicron variants [1, 3, 4], antibody levels measured against Omicron variants were greatly reduced [8, 15].

Collection of DBS for SARS‐CoV‐2 serology was performed as part of a COVID‐19 vaccine effectiveness study using a test‐negative design. Finger stick blood collection was widely acceptable and provided acute‐phase specimens from pediatric and adult outpatients without requiring venipuncture. We used a Tetracore multiplex bead assay that was validated by the manufacturer with sera and standardized in our laboratory for bAb quantification from DBS [13, 14]. While the use of plasma in serologic assays has shown a lower limit of antibody detection [10], paired DBS specimens collected during acute illness and 3–8 weeks after illness may be adequate for assessing antibody responses following natural infection. In this study, paired DBS specimens were tested using the same multiplex bead assay to estimate absolute increase in bAb concentrations. Paired specimens also facilitate comparison between humoral responses in individual patients, including those with vaccine breakthrough infections. Vaccination status and preexisting antibody levels may affect antibody response by altering viral load in early illness [8, 16]. Measurement of immune response to infection and antibody levels after convalescence could improve understanding of vaccine breakthrough cases and hybrid immunity [17].

The study had several limitations. The serologic assay used in our study contained ancestral SARS‐CoV‐2 antigens, and concentrations were converted to binding antibody units using WHO international serum standards from early in the COVID‐19 response. Against pre‐Omicron variants, virus neutralization titers and IgG antibody concentrations were associated with protection; however, antibody binding concentrations and neutralization activity were lower against Omicron variants [2, 8, 18]. In addition, anti‐N bAb seropositivity cut‐off values were based on mean fluorescence intensity using serum standards rather than DBS [9, 13]. All case patients included in this study had mild illness; baseline antibody levels and immune responses may differ among patients with severe or prolonged SARS‐CoV‐2 infection [19]. Among patients with past infection, initial anti‐N bAb concentrations may have waned below the seropositivity thresholds [20, 21, 22]. Finally, this analysis included only four unvaccinated case patients, limiting ability to compare unvaccinated infections and reinfections with vaccine breakthrough infections.

Serologic assays that quantify anti‐SARS‐CoV‐2‐specific antibody levels during and following acute infection may provide information on incidence and recency of infection. As new SARS‐CoV‐2 variants emerge, frequent updates to serologic antigens will be needed to quantify binding IgG antibody levels that correlate with immune protection [15]. Observational studies will be critical for evaluating immune responses to COVID‐19 vaccination and infection.

## Author Contributions

**Ryan Sandford:** methodology, conceptualization, writing–original draft, validation, visualization, formal analysis, investigation. **Ruchi Yadav:** methodology, formal analysis, data curation. **Emma K. Noble:** formal analysis, conceptualization, data curation, writing–review and editing. **Kelsey Sumner:** writing–review and editing, conceptualization, investigation, supervision. **Devyani Joshi:** data curation, writing–review and editing. **Sara Y. Tartof:** writing–review and editing, investigation. **Karen J. Wernli:** investigation, writing–review and editing. **Emily T. Martin:** investigation, writing–review and editing. **Manjusha Gaglani:** investigation, writing–review and editing. **Richard K. Zimmerman:** investigation, writing–review and editing. **H. Keipp Talbot:** investigation, writing–review and editing. **Carlos G. Grijalva:** investigation, writing–review and editing. **Edward A. Belongia:** investigation, writing–review and editing. **Christina Carlson:** funding acquisition, data curation, writing–review and editing. **Melissa Coughlin:** writing–review and editing, supervision, methodology, conceptualization. **Brendan Flannery:** supervision, writing–review and editing, methodology, conceptualization, investigation, funding acquisition, formal analysis, visualization, validation. **Brad Pearce:** supervision, conceptualization, methodology, writing–review and editing. **Eric Rogier:** supervision, formal analysis, investigation, conceptualization, funding acquisition, methodology, writing–review and editing.

## Disclosure

The findings and conclusions in this report are those of the authors and do not necessarily represent the official position of the Centers for Disease Control and Prevention. All authors have reviewed and approved this version of the manuscript.

## Conflicts of Interest

Dr. Zimmerman reports grants from CDC, during the conduct of the study, and grants from Sanofi Pasteur, outside the submitted work. Dr. Grijalva reports other from CDC, grants from NIH, other from FDA, grants and other from AHRQ, other from Merck, and other from Syneos Health, outside the submitted work. Dr. Talbot reports grants from CDC, during the conduct of the study. All other authors report no conflicts of interest.

2

### Peer Review

The peer review history for this article is available at https://www.webofscience.com/api/gateway/wos/peer‐review/10.1111/irv.13339.
